# Fundamental differences in physiology of *Bordetella pertussis* dependent on the two-component system Bvg revealed by gene essentiality studies

**DOI:** 10.1099/mgen.0.000496

**Published:** 2020-12-09

**Authors:** Thomas Belcher, Iain MacArthur, Jerry D. King, Gemma C. Langridge, Matthew Mayho, Julian Parkhill, Andrew Preston

**Affiliations:** ^1^​ Milner Centre for Evolution and Department of Biology and Biochemistry, University of Bath, Claverton Down, Bath, UK; ^2^​ Wellcome Trust Sanger Institute, Hinxton, Cambridge, UK; ^†^​ Present address: Institute Pasteur Lille, Lille, France; ^‡^​ Present address: Quadram Institute, Norwich, UK; ^§^​ Present address: Department of Veterinary Medicine, University of Cambridge, Cambridge, UK

**Keywords:** Bvg, *Bordetella*, essential genes, TraDIS

## Abstract

The identification of genes essential for a bacterium’s growth reveals much about its basic physiology under different conditions. *Bordetella pertussis *, the causative agent of whooping cough, adopts both virulent and avirulent states through the activity of the two-component system, Bvg. The genes essential for *B. pertussis* growth *in vitro* were defined using transposon sequencing, for different Bvg-determined growth states. In addition, comparison of the insertion indices of each gene between Bvg phases identified those genes whose mutation exerted a significantly different fitness cost between phases. As expected, many of the genes identified as essential for growth in other bacteria were also essential for *B. pertussis *. However, the essentiality of some genes was dependent on Bvg. In particular, a number of key cell wall biosynthesis genes, including the entire *mre*/*mrd* locus, were essential for growth of the avirulent (Bvg minus) phase but not the virulent (Bvg plus) phase. In addition, cell wall biosynthesis was identified as a fundamental process that when disrupted produced greater fitness costs for the Bvg minus phase compared to the Bvg plus phase. Bvg minus phase growth was more susceptible than Bvg plus phase growth to the cell wall-disrupting antibiotic ampicillin, demonstrating the increased susceptibility of the Bvg minus phase to disruption of cell wall synthesis. This Bvg-dependent conditional essentiality was not due to Bvg-regulation of expression of cell wall biosynthesis genes; suggesting that this fundamental process differs between the Bvg phases in *B. pertussis* and is more susceptible to disruption in the Bvg minus phase. The ability of a bacterium to modify its cell wall synthesis is important when considering the action of antibiotics, particularly if developing novel drugs targeting cell wall synthesis.

## Data Summary

Sequence data files are available from the European Nucleotide Archive (https://www.ebi.ac.uk/ena), with accession numbers ERS2490348 (Bvg+ phase data) and ERS2490349 (Bvg− phase data).

**Impact Statement**

This study identifies differences in gene essentiality for *B. pertussis *, dependent on the activity of the key Bvg regulatory system. The role of Bvg in regulating the pathogenicity of *B. pertussis* is well established. Here we demonstrate a role in fundamental physiology and surprising differences in cell wall synthesis between Bvg phases. The use of functional genomics to identify the genetic bases for key traits has greatly enhanced our understanding of genotype–phenotype links. Here, we demonstrate that the role of key genes can be conditional, in this case dependent on the activity of a two-component regulatory system. We identify cell wall biogenesis as a process that appears to differ in *B. pertussis* between Bvg states. This suggests novel links between fundamental *B. pertussis* cell growth processes and its pathogenicity and virulence. Our understanding of the role of bacterial physiology, including metabolism in bacterial infection, has expanded significantly in recent years. Elucidating the role of Bvg in *B. pertussis* in regulating cell wall biogenesis will deepen this understanding.

## Introduction

### Essential genes

Allelic replacement mutagenesis is a cornerstone of the study of bacterial pathogenicity. A defined mutation is constructed to produce a strain that is identical to wild-type (WT) except for the loss of the targeted gene. Comparison between the phenotypes of the WT and mutant reveals differences in behaviour that are ascribed to the loss of the targeted gene from the mutant. In this way, the genetic bases of numerous traits have been established and targeted mutagenesis has been used to perturb traits to understand them. However, genes that are essential for the viability of a bacterium cannot be studied in this way because, by definition, the bacteria cannot withstand a knock-out mutation in these genes and remain viable. Often it is not easy to establish that a gene is essential, as the failure to obtain viable mutants is a negative screen and it is not obvious if this failure is because the gene is essential or because the many steps required to achieve the necessary homologous recombination have failed. There is great interest in defining the essential genes of bacteria as part of understanding gene function and genetic and pathway networks [1]. This identifies the core processes required by the bacterium for cell growth, maintenance and division, and can reveal important information about the basic physiology and anatomy of the cell, such as particular metabolic pathways used. Furthermore, identification of processes not absolutely required by the bacterium for viability can be just as informative. Currently, there is great interest in identifying genes that are essential for the viability of bacterial pathogens, as these represent potential targets for the design of novel antimicrobials to combat the relentless rise of antimicrobial resistance [2].

### TraDIS/TnSeq

Post-genomic techniques have proved to be powerful, high-throughput approaches to dissecting the genetic bases for numerous different traits. In particular, transposon-directed insertion sequencing (TraDIS), or TnSeq, is well suited to identifying the essential genes for bacterial viability for whatever conditions the screen has been performed under [3, 4]. A large-scale, high-density library of transposon mutants is generated. These mutants are pooled and genomic DNA is extracted. Utilizing next-generation sequencing, the boundaries between the transposon insertions and neighbouring DNA are sequenced and mapped to the corresponding genome sequence. This identifies the insertion sites for all of the different transposon mutants recovered precisely. If the density of insertions is sufficiently high, then there is high confidence that any genes for which no insertions are observed are those genes that cannot be mutated by insertion to produce a viable mutant, i.e. are the essential genes.

### B. pertussis

*B. pertussis* is the primary causative agent of whooping cough, or pertussis. A well-documented resurgence of pertussis has been observed in many countries in recent years [5]. This has been attributed to several factors, notably a switch from whole-cell to acellular pertussis vaccines in many developed countries during the early 2000s [5]. These vaccines appear to protect infants from serious disease, but induce immunity that wanes more quickly than that induced by whole-cell vaccines, and do not prevent colonization to the same degree as whole-cell vaccines. Combined, it is thought that there is increased circulation/transmission of *B. pertussis* in countries using acellular vaccines. In addition, there are signatures of vaccine-induced selection among *B. pertussis* and it is feared that this selection is favouring the emergence of lineages that are less well controlled by vaccination [6, 7].

### Bvg

The *Bordetella* contain a two-component regulator, Bvg, comprising an inner-membrane histidine kinase, the sensor, BvgS; and the response regulator, BvgA [8]. Two-component systems sense stimuli, often from the bacterium’s environment, and respond to them by regulating changes in gene expression patterns through the activity of the response regulator, which usually comprises transcriptional regulators. Bvg directs *B. pertussis* through a spectrum of different phenotypes. The phenotypes at the extremes of this spectrum are the best characterized. In the absence of stimulus, the levels of phosphorylated BvgA, and thus its transcriptional activating activity, are low. Many of the genes implicated in *B. pertussis* infection, including toxins, adhesins and immune evasion factors, are expressed at very low levels (if at all), whereas other genes are expressed at high levels. This state is referred to as the Bvg minus (Bvg−) phase. On sensing stimulus, activated BvgS phosphorylates BvgA, which triggers its transcriptional activating activity. Through binding to the promoters of target genes, BvgA enhances the transcription of many virulence factors, and also represses a number of the genes that were highly expressed in the Bvg− phase. This virulence-competent state is referred to as the Bvg plus (Bvg+) phase. Bvg transitions *B. pertussis* through a spectrum of states, dependent on the magnitude and duration of the stimulus sensed by BvgS. Initial studies using microarrays identified 291 *B. pertussis* genes differentially regulated by Bvg [9]. RNAseq analysis has identified around 550 genes whose expression is changed by at least 2-fold between Bvg+ and Bvg− phase conditions [10], comprising 14 % of the *B. pertussis* Tohama I gene repertoire. Thus, Bvg regulates the expression of a major portion of *B. pertussis* genes. In addition to genes involved in infection, Bvg appears to regulate many other processes, including metabolism. For example, the Bvg− phase is characterized by a faster growth rate than the Bvg+ phase [11]. Thus, Bvg is regarded as a master regulator of *B. pertussis,* directing it between very different phenotypes. The stimuli sensed by Bvg are unclear, but *in vitro* the system responds to temperature in that *B. pertussis* growing at 37 °C are in the Bvg+ phase, whereas they transition to the Bvg− phase at temperatures below around 27 °C. Also, the Bvg− phase is induced by growth at 50 mM MgSO_4_, even at 37 °C.

Previously, the *B. pertussis* genes essential for growth *in vitro*, and those conditionally essential *in vivo* in a mouse model of infection, were defined [12]. Here we define the genes essential for growth of *B. pertussis in vitro* under both Bvg+ and Bvg− phase conditions. As expected, many of the core processes involved in bacterial growth and replication were identified as essential. However, a surprising number of genes were identified as conditionally essential, dependent on Bvg phase. Furthermore, our use of two different conditions enabled the identification of genes that are not essential, but whose mutation decreases the fitness of *B. pertussis* in a Bvg-dependent manner. Our data identified cell wall biogenesis as a process whose disruption is less well tolerated by Bvg− phase *B. pertussis* compared to Bvg+ phase. The ability of Bvg+ *B. pertussis* to grow despite the mutation of a range of key cell wall biosynthesis genes suggests that this fundamental process differs significantly between Bvg phases.

## Methods

### Bacteria and plasmids

*B. pertussis* BP536 is a streptomycin-resistant clone of Tohama I. BP536 was grown on charcoal agar at 37 °C for 3 days. Bvg− phase conditions were achieved by plating on charcoal agar containing 50 mM MgSO_4_ or 16 mM nicotinic acid. *Escherichia coli* ST18 was used a donor strain for conjugations [13]. It was grown in Luria–Bertani (LB) broth with shaking or on LB agar at 37 °C. ST18 is auxotrophic for aminolevulinic acid (ALA) and thus media were supplemented with 50 mM ALA. *E. coli* 5 alpha (NEB, Hitchin, UK) were used for routine cloning and plasmid maintenance. Selection of strains carrying plasmid pBam1 [14] or ep1 was achieved by supplementing media with 50 μg ml^−1^ kanamycin. pBBR1MCS [15] was used as a shuttle vector to carry a plasmid-borne copy of *mreB* in *B. pertussis *.

### DNA manipulations

Routine DNA manipulations were performed using reagents from NEB or Qiagen (Manchester, UK) according to the manufacturer’s instructions.

### Construction of ep1

Plasmid pBAM1 (14) was modified by the addition of additional *PmeI* restriction endonuclease recognition sites adjacent to the mosaic ends that are recognized by the transposase TnpA, to produce plasmid ep1. This enables cleavage of plasmid-derived DNA to prevent sequencing from plasmid that might be purified from conjugation plates. *PmeI* cuts only once in the *B. pertussis* BP536 genome (within BP0823).

### Construction of transposon mutant libraries

*B. pertussis* BP536 were conjugated with *E. coli* ST18 ep1, as described previously [16]. Conjugants were selected by plating onto charcoal plates supplemented with 50μg ml^−1^ kanamycin and incubation at 37 °C for 3 days. For collection of high-density transposon mutant libraries, nine independent conjugations were performed, with each plated onto multiple 140 mm diameter agar plates. Colonies were recovered into phosphate-buffered saline (PBS) and processed immediately for genomic DNA extraction using the Gentra Pure Yeast/Bact kit (Qiagen, Manchester, UK). For this, portions of each resuspension were mixed in proportion to the number of colonies obtained from the conjugation to give two separate pools of genomic DNA for Bvg+ phase and Bvg− phase conjugants. The purified DNA was digested with PmeI to prevent sequencing from ep1 plasmid DNA that was harvested from dead donor *E. coli* present on the conjugation plates.

### DNA sequencing of transposon mutant libraries

Two micrograms of genomic DNA was fragmented by Covaris to an average size of ~300 bp. Following end repair and ‘A’ tailing, a modified Illumina adapter, synthesized and annealed by IDT using oligonucleotides SplA5_top and SplA5_bottom (Table 1), was ligated to the fragments for 40 min at 20 °C. Ligated fragments were cleaned using Ampure XP beads (Beckman Coulter, High Wycombe, UK) with a beads-to-sample ratio of 0.8 to 1. PCR enrichment of fragments containing transposon was performed using primers homologous to each end of the transposon (EP1 5′PCR or EP1 3′PCR) in conjunction with an adapter-specific primer containing an index tag (SplAP5.x). PCR products were cleaned using Ampure XP beads as before, quantified by qPCR and then pooled. PhiX library was added to a level of 6 %. The pooled libraries were sequenced on a HiSeq 2500 using a specially modified recipe to overcome difficulties generated by the monotemplate transposon sequence. Briefly, a transposon-specific sequencing primer (EP1 5′seq or EP1 3′seq) anneals to the transposon 10 bases away from the junction between transposon and *B. pertussis* chromosome. Sequencing takes place with no imaging for the first 10 cycles followed by imaging for the next 50 cycles. This gives a 50 bp genomic DNA read. The template is denatured and the same sequencing primer is reannealed and 10 cycles of sequencing take place to give a 10 bp transposon read. To reduce background, plasmid DNA libraries were digested with *Pme*I at the post-ligation stage for the 3′ transposon ends and a blocking oligo (added during PCR enrichment) for the 5′ transposon ends.

**Table 1.** Primers used in this study

Primer	Sequence (5′ to 3′)
SplA5_top	G*AGATCGGTCTCGGCATTCCTGCTGAACCGCTCTTCCGATC*T
SplA5_bottom	/5Phos/G*ATCGGAAGAGCGGTTCAGCAGGTTTTTTTTTTCAAAAAAA*A
EP1 5′PCR	AATGATACGGCGACCACCGAGATCTACACTTATTGTTCATGATGATATATTTTTATCTTGTGC
EP1 3′PCR	AATGATACGGCGACCACCGAGATCTACACGCAGGTCGACTCTAGAGGATCCCC
EP1 5′seq	TAACATCAGAGATTTTGAGACACAAGACGTCAGATGTGTA
EP1 3′seq	GCGGCCTAGGCGGCCTTAATTAAAGATGTGTA
5′plasmid blocking oligo	CATCAGATTCTGGAAAACGGGAAAGGTTCCGTTCAGGACGCTACTTGTGTAGTTTAAACCAGCTGG†
SplAP5.1	C*AAGCAGAAGACGGCATACGAGATAACGTGATGAGATCGGTCTCGGCATTC*C
SplAP5.2	C*AAGCAGAAGACGGCATACGAGATAAACATCGGAGATCGGTCTCGGCATTC*C
SplAP5.3	C*AAGCAGAAGACGGCATACGAGATATGCCTAAGAGATCGGTCTCGGCATTC*C
SplAP5.4	C*AAGCAGAAGACGGCATACGAGATAGTGGTCAGAGATCGGTCTCGGCATTC*C
SplAP5.5	C*AAGCAGAAGACGGCATACGAGATACCACTGTGAGATCGGTCTCGGCATTC*C
SplAP5.6	C*AAGCAGAAGACGGCATACGAGATACATTGGCGAGATCGGTCTCGGCATTC*C
SplAP5.7	C*AAGCAGAAGACGGCATACGAGATCAGATCTGGAGATCGGTCTCGGCATTC*C
SplAP5.8	C*AAGCAGAAGACGGCATACGAGATCATCAAGTGAGATCGGTCTCGGCATTC*C
SplAP5.9	C*AAGCAGAAGACGGCATACGAGATCGCTGATCGAGATCGGTCTCGGCATTC*C
SplAP5.10	C*AAGCAGAAGACGGCATACGAGATACAAGCTAGAGATCGGTCTCGGCATTC*C
SplAP5.11	C*AAGCAGAAGACGGCATACGAGATCTGTAGCCGAGATCGGTCTCGGCATTC*C
SplAP5.12	C*AAGCAGAAGACGGCATACGAGATAGTACAAGGAGATCGGTCTCGGCATTC*C
mreBLF	AAAAGGTCTCTCGAGATTCGGGGGCGTTGG
mreBLR	AAAAGGTCTCCATGTGCATGGGAGCTCAGCTAGATTC
mreBRF	AAAAGGTCTCAGGTCTGAGCCTGTCTCGCG
mreBRR	AAAAGGTCTCGAACTGGGCGGCTCGTACAGC
KanF	AAAAAAGGTCTCCACATGACGTCTTGTGTCTCAAAATCTC
KanR	AAAAAAGGTCTCAGACCTTAGAAAAATTCATCCAGCATC

*Phosphorothioate group modification.

‡Dideoxy base.

### Analysis of DNA sequence

Sequencing data were analysed as described previously [3] to process reads, map them to the Tohama I reference genome sequence, define the number of unique insertions and the insertion index for each gene, predict the essentiality of each gene, and compare mutant frequencies between the Bvg+ and Bvg− phase conditions. Briefly, an insertion index for each gene was calculated by dividing the number of unique insertion sites within the gene by gene length (to normalize for gene length). The distribution of insertion indices was bimodal with a narrow peak corresponding to essential genes without insertions and a broader distribution for genes able to tolerate insertions. Fitting gamma distributions to these two modes enabled calculation of log_2_ likelihood ratios (LLRs) between the two modes. An LLR of <−2 was used to assign a gene as essential (indicating that it was four times more likely to be essential than non-essential), while an LLR of >2 was used to assign as non-essential. Genes falling between these values were classed as ambiguous in terms of their essentiality. Identification of genes for which there were significantly different numbers of read between Bvg phases was conducted as described previously [3].

### Availability of data and materials

Sequence data files are available from the European Nucleotide Archive (https://www.ebi.ac.uk/ena), with accession numbers ERS2490348 (Bvg+ phase data) and ERS2490349 (Bvg− phase data).

### Antibiotic susceptibility testing

The minimum inhibitory concentrations (MICs) of Bvg− and Bvg+ phase *B *. *pertussis* for antibiotics were determined using E-test strips (BioMérieux, Basingstoke, UK). BP536 was grown on charcoal agar and resuspended in PBS to an OD_600_=0.2. Then 100 μl of this suspension was plated onto charcoal agar or charcoal agar supplemented with 50 mM MgSO_4_. Once the plates were dry, an E-test strip was placed centrally and the plates were incubated at 37 °C for 72 h. MICs were read as described in the manufacturer’s literature (http://www.biomerieux.co.uk/sites/subsidiary_uk/files/etest-reading-guide-aerobic-bacteria.pdf.) The average and standard errors of MICs for triplicate samples were calculated and significance was determined using a *t*-test.

### Construction of *mreB* mutant

Allelic exchange mutagenesis was used to delete the *mreB* gene and replace it with a kanamycin resistance cassette. Regions flanking *mreB* and a kanamycin resistance gene cassette were amplified by PCR (Q5 high-fidelity polymerase, NEB) using the primers indicated in Table 2. They were cloned using Golden Gate cloning into plasmid pCR8 (Invitrogen, Loughborough, UK) modified for Golden Gate cloning. This cloned region was subcloned into the allelic exchange vector pSS4940 using Gateway technology (Invitrogen). The resulting plasmid was transformed into the conjugation donor *E. coli* strain ST18, and introduced into *B. pertussis* by conjugation as described previously [16]. Conjugants were selected on charcoal agar supplemented with 50 mM MgSO_4_ and 30 µM gentamycin at 37 °C for 3–4 days. Individual clones were passaged on this media prior to passaging on charcoal agar supplemented with 50 µg ml^−1^ kanamycin at 37 °C for 3–4 days to select for allelic exchange mutants. Individual clones were analysed using PCR to confirm the replacement of the *mreB* gene by the kanamycin resistance cassette.

**Table 2.** Functional categories of essential and fitness-affected genes

	Unconditional	Bvg+ only	Bvg− only
Amino acid biosynthesis	19	0 (1)	9 (4)
Capsule biosynthesis	0	0 (7)	0 (0)
Cell division	12	1 (3)	2 (3)
Cell wall	54	4 (2)	19 (17)
Chaperone	4	0 (0)	0 (0)
Chemotaxis	0	0 (0)	1 (0)
Cofactor biosynthesis	52	1 (1)	4 (1)
DNA repair	4	3 (0)	2 (1)
DNA replication	16	0 (2)	3 (2)
Electron transport	6	0 (0)	0 (3)
Energy metabolism	23	0 (0)	1 (0)
General metabolism	1	1 (4)	0 (1)
Glycolysis/gluconeogenesis	10	0 (0)	0 (0)
Homeostasis	14	0 (4)	5 (2)
Lipid metabolism	14	0 (1)	1 (2)
Mobile genetic element	0	0 (1)	0 (0)
Nucleotide metabolism	7	0 (0)	0 (0)
Oligosaccharide biosynthesis	0	0 (3)	0 (0)
Pentose phosphate pathway	3	0 (0)	0 (0)
Peptidase	1	0 (0)	0 (0)
Phospholipid metabolism	6	0 (0)	0 (0)
Protein secretion	10	0 (0)	1 (1)
Purine metabolism	6	0 (0)	0 (0)
Pyrimidine metabolism	3	0 (0)	0 (1)
Response regulation	2	0 (1)	1 (1)
RNA metabolism	1	0 (0)	0 (0)
Small molecule transport	2	0 (4)	3 (4)
Stress response	3	0 (2)	0 (1)
TCA cycle	8	0 (0)	6 (0)
Transcription	10	2 (2)	2 (2)
Translation/ribosome structure	98	2 (1)	12 (1)
Unknown	7	5 (11)	7 (4)
Total	396	19 (50)	79 (51)

### Viable counts of *mreB* mutant

WT and *B. pertussis mreB* were grown on charcoal agar. Bacteria were suspended in PBS and serially diluted. Then 100 µl of dilutions were plated onto charcoal agar and charcoal agar supplemented with 50 mM MgSO_4_ or 16 mM nicotinic acid and incubated at 37 °C for 4 days, and the resulting colonies were imaged.

## Results

To create the library of transposon mutants, nine independent conjugations were performed in which the transient expression of Tn5 transposase led to mTn insertions into the *B. pertussis* chromosome. Plasmid ep1 contains a modified mTn5 containing the Tn5 direct repeats flanking a kanamycin resistance cassette. It carries the Tn5 transposase gene, but outside of the repeats on which it acts. Plasmid ep1 cannot replicate in *B. pertussis *, but on delivery into these bacteria by conjugation, the transient expression of the transposase enables transposition of the kanamycin cassette onto the *B. pertussis* chromosome but subsequent loss of the plasmid. Conjugants from each conjugation were selected on agar under both Bvg+ and Bvg− phase conditions. Approximately 490 000 transposon insertion mutants were recovered for each Bvg phase condition. Bvg+ phase and Bvg− phase mutants were recovered separately for each conjugation. Single pools of Bvg+ and Bvg− phase mutants were created by combining colonies obtained from each conjugation in proportion to the number of colonies recovered in each, and genomic DNA was extracted for each pool and subjected to sequencing. For the Bvg+ and Bvg− mutant libraries, 300 581 and 316 281 unique insertion sites were identified, corresponding to an average of an insertion every 13.6 and 12.9 bp, respectively, across the 4 086 189 bp *B. pertussis* genome, i.e. very high-density transposon libraries had been recovered.

**Fig. 1.**
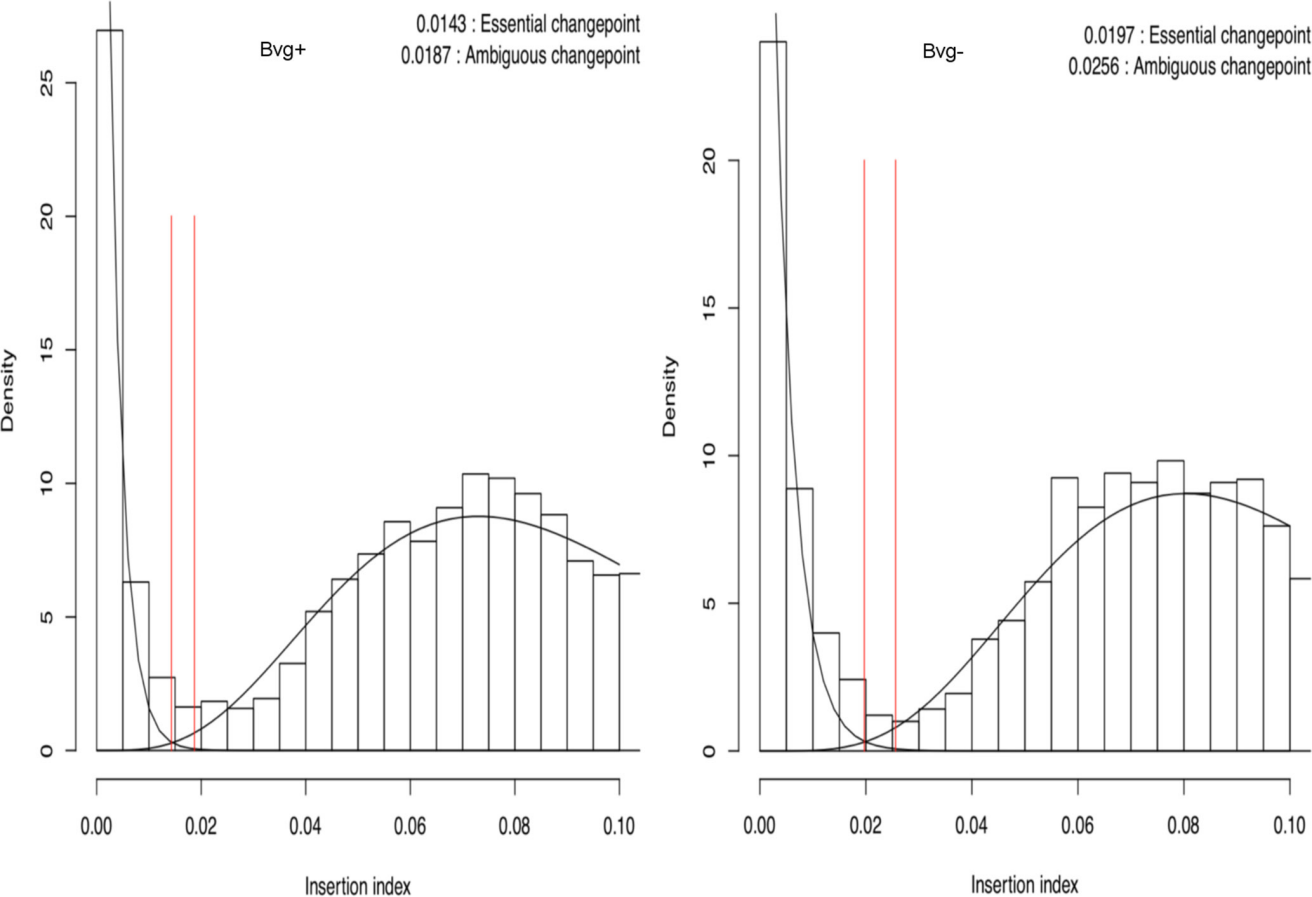
Gamma-fitted plots of the frequency distribution of the insertion indices for Bvg+ and Bvg− phase transposon mutant libraries. The bimodal distribution highlights essential genes (left peaks) and non-essential genes (right peaks). The cutoff values of insertion indices for designating genes as essential or whether their essentiality is ambiguous are shown.

Using a previously described approach [3, 17], the genes that are essential for growth under Bvg+ and Bvg− phase conditions were calculated. Due to the uncertainty regarding the ability of bacteria to tolerate insertions in the termini of essential genes, here insertions in the terminal 10 % of each gene were discounted for the purpose of essential gene identification. The number of unique insertion sites within each gene was normalized by gene length to give an insertion index for each gene. Plotting the frequency distribution of insertion indices produces a bimodal distribution representing essential and non-essential genes. This was used to calculate a likelihood ratio for whether each gene was more likely to be in the essential gene peak of the distribution and a cutoff value was calculated to define whether a gene was considered essential or non-essential. The gamma fits of these distributions for insertion indices for the Bvg+ and Bvg− phase mutants are shown in Fig. 1.

Three hundred and ninety-six genes were identified as essential, in that they were required for growth of both Bvg+ and Bvg− phase *B. pertussis* under the conditions tested here (Table S1, available in the online version of this article). A further 26 genes were ambiguous for essentiality for each of the Bvg+ and Bvg− phases (Table S2), in that their insertion indices fell between the two peaks in the distribution of frequencies.

The broad functional categories of these genes are listed in Table 2. Three broad functional classes are richly represented among these genes, comprising 203 of the 396 essential genes: translation/ribosome structure, cell wall and cofactor biosynthesis. These processes are also richly represented in the essential gene lists of many other bacteria, highlighting the importance of these core processes for cellular viability and growth, and the large number of genes involved in these processes. Also, as expected, key metabolic genes were identified as essential. For example, *B. pertussis* does not possess a functional glycolytic pathway and does not metabolize sugars as a carbon source [18, 19]. It is assumed that gluconeogenesis is used to synthesize the sugar components of *B. pertussis* via the metabolism of glutamate, the main carbon source in the media used here [20]. In agreement with this, every gene encoding a gluconeogenesis enzyme is essential.

Table 2 The number of genes within different functional categories identified as essential under both Bvg+ and Bvg− phase conditions (unconditional), or in only the Bvg+ or Bvg− phase. The number of genes within each functional category that although not essential produce a statistically different fitness cost between phases is in parentheses.

There were just seven genes identified as being essential for which involvement in a particular cellular process could not be predicted. These represent potentially novel core processes in *B. pertussis* or novel functions in previously characterized core processes. Homologues of all of the predicted proteins encoded by these genes are present in other bacteria. Of these seven predicted proteins, only one contains strong homology to conserved domains as revealed by blastp homology searches. On the basis of these results, BP2815 is a putative glycosyl hydrolase. However, the substrate, and thus cellular function, of BP2815 is unknown.

Of particular interest was the identification of genes that were only essential in either the Bvg+ or the Bvg− phase, i.e. conditionally essential genes. Nineteen genes were identified as being essential in the Bvg+ phase but not the Bvg− phase (Table S1). Six of these genes were, however, described in the ambiguous category in the Bvg− phase, meaning that it is not definitively clear as to whether they are conditionally essential. For those genes that have been either characterized or for which a putative function prediction is possible, a variety of functions are evident. There is not a particular functional category that is richly represented within this group and thus there is not a specific process identifiable that is only essential in the Bvg+ phase.

In contrast, there are 79 genes that are only classified as essential in the Bvg− phase. Ninetheen of these are classed as ambiguous in Bvg + phase bacteria, but this still identifies a large number of genes as only being essential under Bvg− phase conditions. Among these genes, 3 broad functional categories stand out: 19 genes are predicted to encode cell wall biosynthesis functions, 12 genes are involved in translation/ribosome structure and 6 genes encode components of TCA cycle enzyme complexes.

Many genes are not essential but mutation of them carries a fitness cost that results in transposon insertions in that gene being recovered at relatively low frequency compared to genes that are completely dispensable for growth under the conditions tested. Statistical comparison of the number of sequence reads mapping to each gene between the Bvg phases enabled the identification of genes whose mutation induces significantly different fitness costs between Bvg phases. This analysis identified 50 genes for which insertion mutants were recovered at lower frequency for the Bvg+ phase and 51 genes for which mutants were recovered at a lower frequency for the Bvg− phase (Table S3). Of these genes, only 8 (Bvg+) and 16 (Bvg−) were essential (conditionally or otherwise), demonstrating the power of this approach to extend the analysis of the fitness of mutants between Bvg phases. It is important to note that this does not mean that mutations in conditionally essential genes in the permissive phase do not cause a fitness defect. For some of these genes, their insertion indices in the permissive phase were low (but above the threshold for essentiality), suggesting that while viable mutants were recovered, their numbers were low compared to mutants for truly dispensable genes. However, when comparing the read counts for these genes between the the two phases, as in our analysis of fitness, the difference does not reach significance.

While these data contain a wealth of gene-specific information, overall, there was a strong signal for disruption of cell wall biosynthesis and energy metabolism producing greater fitness costs for *B. pertussis* in the Bvg− phase compared to the Bvg+ phase.

### Cell wall biosynthesis

**Fig. 2.**
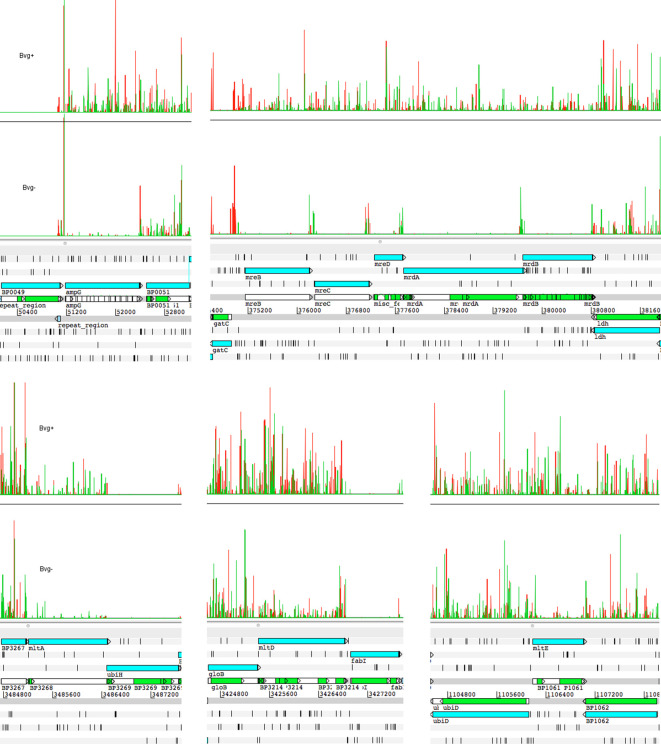
Plots of insertion sites and frequencies in the genes discussed in the text for Bvg+ and Bvg− phase conditions. The height of the lines indicates the frequency of insertions at that site. Green and red distinguish the orientation of the transposon insertion into the chromosome. Plots were generated from mapped read files and visualized in Artemis as described previously [3].

A number of cell wall biosynthesis genes were identified as essential for growth in the Bvg− phase but not the Bvg+ phase. Others were not essential in either phase, but their mutation affected the fitness of Bvg− B. *pertussis* more than it did for the Bvg+ phase *B *. *pertussis *. In particular, genes involved in assembly of new cell wall are represented in this gene set, for example *ampG*, the entire *mre/mrd* operon, d-alanyl-d-alanyl carboxypeptidase DacC, and a number of murein endopeptidases or murein transglycosylases (Table 3, Fig. 2).

**Table 3.** Penicillin-binding proteins and murein lytic transglycosylases of *B. pertussis* and their essentiality/role in fitness

Gene	Protein name	Function	Essential/fitness affected?
BP0102	DacC	d-ala-d-ala carboxypeptidase	Essential Bvg− Fitness Bvg−
BP0377	MrdA (Pbp2)	Cell elongation transpeptidase	Essential Bvg− Fitness Bvg−
BP0905	MrcA (Pbp1A)	dd-transpeptidase	Non-essential
BP1051	DacB	d-ala-d-ala carboxypeptidase	Non-essential
BP1545	DacC2	d-ala-d-ala carboxypeptidase	Non-essential
BP2754	Pbp1C	dd-transpeptidase	Non-essential
BP3028	FtsI	Cell division transpeptidase	Essential Bvg+/−
BP3655	Pbp?	dd-transpeptidase	Non-essential
BP1061	MltE	Murein lytic transglycosylase	Fitness Bvg−
BP3214	MltD	Murein lytic transglycosylase	Fitness Bvg−
BP3268	MltA	Murein lytic transglycosylase	Fitness Bvg−

AmpG functions to recycle peptidoglycan fragments, released during cell growth, back into the cell [21]. *B. pertussis* releases tracheal cytotoxin during culture. TCT is N-acetylglucosaminyl-1,6-anhydro-N-acetylmuramyl-(L)-alanyl-γ-(D)-glutamyl-mesodiaminopimelyl-(D)-alanine and is cytotoxic towards mammalian epithelial cells [22]. Its release from *B. pertussis* is ascribed to *B. pertussis* AmpG being non-, or poorly, functional, as expression of *E. coli ampG* in *B. pertussis* greatly reduced the amount of TCT in culture supernatants [23]. TCT was released by both Bvg+ and Bvg− phase *B. pertussis* [24], but the relative amounts released by the two phases were not noted. Thus, although *B. pertussis* AmpG may have low levels of activity compared to other bacteria, our data suggest that for growth of the Bvg− phase, recycling of peptidoglycan fragments is a factor.

The *mre* locus contains genes encoding proteins commonly annotated as ‘rod shape-determining proteins’. Briefly, these proteins are key components of the cell wall synthesis machinery. Together they coordinate and participate in the insertion of newly synthesized peptidoglycan fragments into the peptidoglycan sheath and this is vital for maintenance of cell shape, cell rigidity and cell growth [25, 26]. Viable mutants of these genes have been constructed in other bacteria. Usually they have altered cell shape, and often they are only able to grow under specific conditions, such as in minimal media [25]. It is striking that none of the genes in this locus are essential for growth in the Bvg+ phase. Transposon insertions were recovered throughout each of these genes in Bvg+ phase conditions (Fig. 2), strongly suggesting that inactivation of each gene gave rise to viable bacteria.

DacC is a penicillin-binding d-alanyl-d-alanyl carboxypeptidase involved in transpeptidation reactions in peptidoglycan biosynthesis. Most Gram-negative bacteria encode several/multiple penicillin-binding proteins (PBPs), suggesting redundancy among their functions or that different PBPs are utilized under different conditions. Analysis of the *B. pertussis* genome sequence identified eight genes encoding putative PBPs (Table 1). One of these was the cell division transpeptidase FtsI, and one was MrdA in the *mre*/*mrd* locus described above. Interestingly, of the other putative dd-transpeptidases, only that encoded by BP0102 was essential, and then only in the Bvg− phase. Another DacC homologue, encoded by BP1545, was identified. It displays little homology to BP0102 DacC at the amino acid level, although it contains strong homology to the DacC conserved domain (COG1686) with a blastp score of 1.49e^−72^. This suggests that BP0102-encoded DacC has a function that is distinct from those of the other putative DD transpeptidases, and that is essential for viability in the Bvg− phase.

BP1061 (MltE), BP3214 (MltD) and BP3268 (MltA) all encode putative murein lytic transglycosylases. These enzymes cleave the glycosidic bond between the MurNAc and GlcNAc residues of peptidoglycan during recycling of the cell wall or biosynthesis of new cell wall regions; during cell growth and division. None were essential, but all three were identified as affecting the fitness of Bvg− phase *B. pertussis* more than Bvg+ phase.

**Fig. 3.**
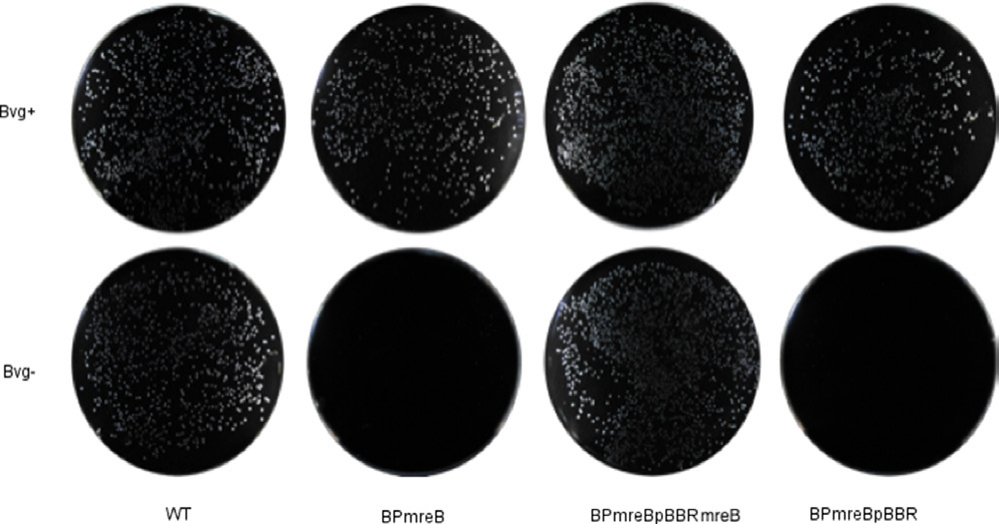
*B. pertussis mreB* is unable to grow under Bvg− phase conditions. Strains were grown on plates under Bvg+ phase conditions. Bacteria were resuspended in PBS and serially diluted, and dilutions were plated onto agar and grown under either Bvg+ or Bvg− phase conditions for 72 h until colonies were clearly visible. All strains grew very similarly under Bvg+ and Bvg− phase conditions, except for the *B. pertussis mreB* mutant (BPmreB), which did not grow under Bvg− phase conditions. BPmreBpBBR, the mreB mutant carrying the shuttle vector pBBR1MCS. BPmreBpBBRmreB, the *mreB* mutation complemented by a copy of *mreB* carried on the shuttle vector pBBR1MCS.

To validate findings made using TraDIS, and to further investigate differences in cell wall biosynthesis between Bvg phases, a defined mutant in *mreB* was constructed. During construction, the mutant strain was only grown in Bvg+ phase conditions, and in doing so viable *mreB* mutants were obtained. These mutants produced normal-looking colonies on charcoal agar after 72 h incubation at 37 °C, as does WT, confirming that *mreB* is not required for the normal growth rate of *B. pertussis* in the Bvg+ phase. The viability of the mutant under Bvg− phase conditions was tested. Serial dilutions of WT and *B. pertussis mreB* grown under Bvg+ phase conditions were plated onto Bvg+ and Bvg− phase condition charcoal agar plates and the number of viable colonies recovered was compared (Fig. 3). Under Bvg+ phase conditions there was no difference between the number of WT and mutant colonies recovered. However, under Bvg− phase conditions, no colonies of the *mreB* mutant were recovered, confirming the TraDIS-derived observation that *mreB* is conditionally essential for the growth of *B. pertussis* on charcoal agar, dependent on Bvg activity. Complementation of the mutation with a plasmid-borne copy of *mreB* restored growth under Bvg− phase conditions, demonstrating that disruption of *mreB* produced this phenotype. It is possible that the lethality of the *mreB* mutation on Bvg− condition plates was due to the high levels of MgSO_4_ used to induce Bvg− phase conditions, possibly via effects on osmotic pressure. However, the same lethality was observed when using nicotinic acid to induce Bvg− phase conditions (data not shown), another known modulator of Bvg. In addition, attempts to construct the *mreB* mutation in a Bvg mutant background were unsuccessful, consistent with knock out of *mreB* being lethal to Bvg− phase bacteria.

**Fig. 4.**
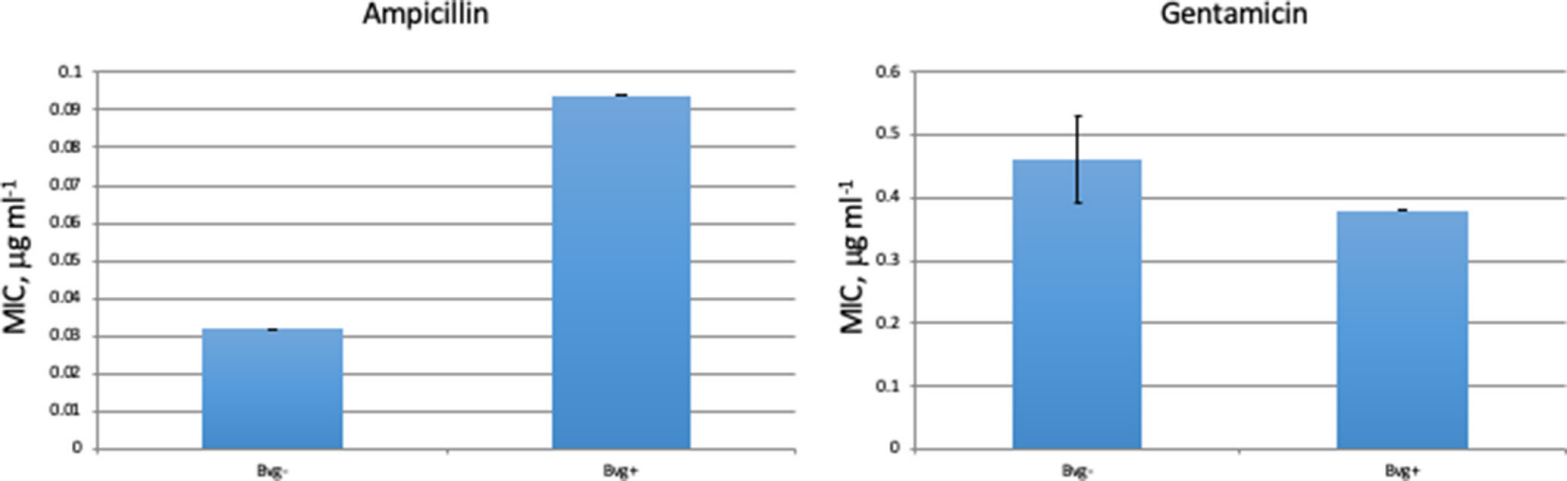
The MIC for ampicillin and gentamicin of *B. pertussis* grown under Bvg+ and Bvg− phase conditions. The graphs show the average and standard error of triplicate samples. Significance was calculated using a *t*-test. *P <0.05.

Together, these data strongly suggest that the growth of Bvg− phase *B. pertussis* is more sensitive to disruption of new cell wall synthesis than is the Bvg+ phase. It was reasoned that the higher sensitivity of Bvg− *B. pertussis* to disruption of cell wall synthesis compared to Bvg+ *B. pertussis* would result in them being more sensitive to cell wall-disrupting antibiotics. The MIC for ampicillin was determined for *B. pertussis* in both phases and was significantly lower for cells growing in the Bvg− phase, i.e. these bacteria were significantly more susceptible to growth inhibition by ampicillin than Bvg+ phase bacteria (Fig. 4). The sensitivity to gentamicin, an inhibitor of protein synthesis, although slightly greater for Bvg+ phase bacteria, was not significantly different to that of Bvg− phase bacteria suggesting that this effect was specific for the cell wall-disrupting activity of ampicillin (Fig. 4).

Mutation of these genes appears to cause different effects on the Bvg phases of *B. pertussis *. Bvg is a global regulator of gene expression in *B. pertussis* and thus these differences might be due to different levels of expression of these genes between the phases. However, these genes are not Bvg-regulated, as determined by RNAseq-mediated analysis of gene expression of Bvg+ and Bvg− phase *B. pertussis* (unpublished data and [10]) and each of the genes was expressed in both Bvg phases. This suggests that the increased sensitivity of the Bvg− phase to mutation of these genes was not due to lack of expression in the Bvg+ phase, but due to different sensitivity of the phases to disruption of the activities in which the proteins encoded by these genes are involved.

### TCA cycle/energy metabolism

Succinate dehydrogenase (SDH) participates in both the TCA and electron transport chain. *B. pertussis* cannot metabolize sugars and preferentially uses glutamate as a carbon source. Thus many biosyntheses in *B. pertussis* stem from metabolism of glutamate via alpha-ketoglutarate and the TCA cycle. Genes encoding all four subunits of SDH were identified as being essential for Bvg− *B. pertussis *, as perhaps would be expected for such a key metabolic enzyme complex. Surprisingly, SDH was not essential for growth of Bvg+* B. pertussis *, although the insertion indices for *sdhA-D* in Bvg+ *B. pertussis* were low. It is not clear how glutamate might be metabolized in the absence of SDH, as metabolism of the α-ketoglutarate through which glutamate-derived carbon enters the TCA requires SDH. In addition, SDH generates FADH_2_, which transfers electrons to the electron transport chain for the generation of the proton motive force and ATP production.

**Fig. 5.**
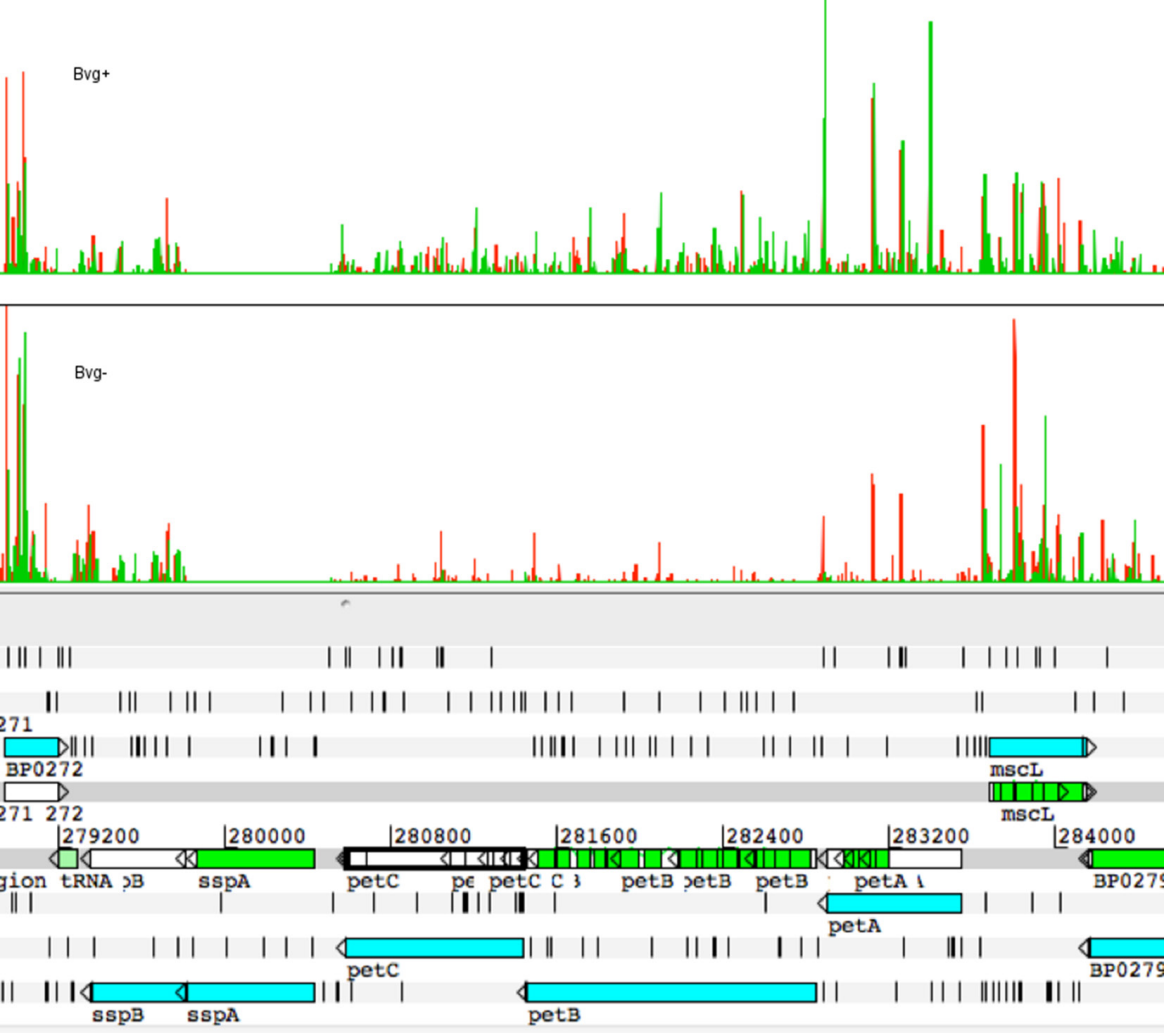
Plots of insertion sites in the *petABC* locus for Bvg+ and Bvg− phase conditions. The height of the lines indicates the frequency of insertions at that site. Green and red distinguish the orientation of the transposon insertion into the chromosome. Plots were generated from mapped read files and visualized in Artemis as described previously [3].

A difference between phases in the effect of mutation of the cytochrome bc complex (complex III) was observed. The insertion indices for *petABC* were significantly lower for Bvg− *B. pertussis* (0.078, 0.053, 0.064) compared to Bvg+ phase (0.183, 0.165, 0.162), *P*=3.88E-09, 3.43E-14, 1.36E-06, respectively (Fig. 5). The cytochrome bc complex is a key link between electron transport and proton translocation for utilizing metabolically derived energy to generate the proton motive force. Most bacteria contain alternative pathways for quinol oxidation that bypass the cytochrome bc complex, but many of these do not have the same energy transduction, and thus the same proton translocation power, as the cytochrome bc complex. The relative lack of sensitivity of Bvg+ *B. pertussis* to mutation of *petABC* suggests that growth in the Bvg+ phase is less reliant on this electron transport complex than growth in the Bvg− phase. These findings suggest that energy metabolism in *B. pertussis* differs between the Bvg phases.

There were an equal number of genes whose insertion index was significantly lower under Bvg+ phase conditions compared to Bvg− conditions. Interestingly, a different set of cell wall biogenesis/cell division genes than those with lower insertion indices under Bvg− conditions were in this group, again suggesting that there are differences in these processes between Bvg phases. Of note, also within this group were a number of genes involved in synthesis and export of the *Bordetella* extracellular polysaccharide (EPS), often referred to as the *Bordetella* capsule. Although relatively poorly characterized, the genes in this locus are maximally expressed under Bvg− phase conditions, although basal levels of transcription are observed under Bvg+ phase conditions [9]. Recently, upregulation of the capsule locus was observed in *B. pertussis* during infection of mice, conditions under which *B. pertussis* had been assumed to be in the Bvg+ phase [27]. The same study identified an interaction between components of the EPS export apparatus and BvgS, and mutation of EPS export genes caused wide-ranging effects on the expression of a number of Bvg-regulated genes. While interesting, it is unclear why mutation of the capsule locus causes fitness costs in the Bvg+ phase *in vitro*, conditions under which the locus is only expressed at low levels, whereas there is no apparent defect under Bvg− phase conditions, in which there is high-level expression of the locus.

## Discussion

Previously, the genes essential for the growth of *B. pertussis in vitro* and on Bordet–Gengou agar and those conditionally essential for survival of *B. pertussis in vivo* in a mouse model of infection were defined [12]. Here, we focused on the effect of the global regulator of *B. pertussis* virulence, the Bvg two-component system, and identified genes that were conditionally essential for *in vitro* growth of *B. pertussis *, dependent on the activity of Bvg. Bvg is hypothesized to sense stimuli that signal to *Bordetella* that they have entered a host, as switching from ambient temperature to 37 °C switches them from the Bvg− to the Bvg+ phase, which is characterized by high-level expression of many of the proteins (e.g. adhesins, toxins) involved in infection. In this way, Bvg has been regarded as a switch that regulates the transition between virulent and avirulent states. Several studies have identified that Bvg regulates the expression of a large number of genes (for example, 550 genes [10]), including those maximally expressed in either the Bvg+ or the Bvg phase. A spectrum of states exists between these two extremes, with some genes maximally expressed in intermediate states. The role of Bvg in the regulation of virulence/infection in the bordetellae has been well studied. Much less is known regarding Bvg− regulation of basic physiology. Bvg− regulation of growth rate has been observed. For example, during laboratory culture, compared to Bvg+ phase *B *. *pertussis *, Bvg− phase *B. pertussis* exit lag phase earlier, grow at a faster rate during exponential growth and reach higher biomass final yields [11], but the genetic basis for this is unknown.

We identified 396 genes that were unconditionally essential for the growth of strain BP536, compared to 609 genes defined as essential for strain UT-25 [12]. As expected, many of the genes identified here were also essential for UT-25 (Table 2). Thirty-seven genes identified as essential for BP536 were not essential for UT-25, whereas 134 genes essential for UT-25 were dispensable for growth of BP536. The two studies used different laboratory agars for growth (charcoal vs Bordet–Gengou), but both are similar, utilizing peptone as the major carbon source for growth, and as a source of starch (not metabolized by *B. pertussis *), to complex small, hydrophobic inhibitory molecules. Here, Tn5 was used, whereas Gonyar *et al*. used a Mariner-based transposon. However, in both cases high-density transposon libraries were recovered, suggesting saturation of the genome. Thus, these differences in gene essentiality may represent differences between the two strains in their requirements for growth. Of particular note, two loci were identified as essential in UT-25 but not BP536, comprising 20 of the 23 genes between BP0911–BP0934 and 17 of the 19 genes between BP1158–BP1176. The functions of genes in these regions are unknown, but many appear to encode transport and metabolic functions. Fascinatingly, the genes BP0910–0934 represent one of the known regions of difference between strains, in that this region is absent from many *B. pertussis* strains [28, 29]. Thus, it is surprising that it is essential for strain UT-25, when this region is clearly dispensable to many other strains. No loci of contiguous genes were identified as essential in BP536 but not UT-25, and it is possible that differences in the sensitivity of approaches used to identify essential genes contributes to some of these differences.

Here, we identified some surprising differences in the effect of mutations in genes involved in certain core processes, dependent on the activity of Bvg. Seventy-nine genes were identified as only being essential in the Bvg− phase, not the Bvg+ phase. Analysis of the predicted functions of these genes identified cell wall biogenesis, cofactor biosynthesis and translation as processes for which more genes were essential in the Bvg− phase compared to Bvg+.

Identification of genes whose mutation is not lethal, but appears to affect the fitness of the bacterium, expands this observation. Of the 51 genes whose mutation significantly affects the fitness of Bvg− phase bacteria but not Bvg+ phase, 35 encode functions involved in these same 3 processes, compared to only 14 genes for the Bvg+ phase. Among these genes are those considered central to cell wall biogenesis, for example the *mreBCDmrdAB* operon, which encodes proteins that coordinate the insertion of newly synthesized peptidoglycan fragments into the existing cell wall. In some other bacteria, strains carrying knock-out mutations of these genes are viable, but only under certain conditions, such as nutrient limitation. For example, *E. coli mreB* mutants are spheroid rather than the WT rod shape [25]. Interestingly, for *Bacillus subtilis mre* mutants, the growth and shape defects usually associated with mutations in these genes were completely suppressed by supplementing the growth media with magnesium, presumably through stabilization of the cell wall [30]. In *B. pertussis *, mutations in this locus appear remarkably well tolerated under Bvg+ phase conditions, as reasonable numbers of insertions in these genes were counted in the mutant library, and insertions were recovered throughout each gene of the locus. The mutants were recovered from plates at a single time point, 72 h after plating, which would be considered normal growth for *B. pertussis* and suggests that these mutants grew at normal rates. We constructed a defined knock-out mutation of *mreB* in *B. pertussis *. Full characterization of the phenotypes of this mutant, and of other *mre/mrd* mutants, is on-going, but it confirmed that *mreB* was not required for the WT growth rate of Bvg+ phase *B *. *pertussis *, whereas this mutant was non-viable under Bvg− phase conditions despite the Bvg− phase being induced by the addition of Mg^2+^ to the medium.

A number of studies have identified the essentiality of genes as being conditional through changing growth conditions, often using different media compositions or growth in the presence of different stressors. Here, the conditional essentiality of genes involved in core processes is demonstrated, dependent on the activity of a regulator of gene expression. It is recognized that Bvg state was manipulated by the addition of MgSO_4_ to the agar, but this was the only difference in the growth conditions between the Bvg phases.

It might be expected that Bvg would thus influence the essentiality of genes, as strong downregulation of genes (switching off expression) in one Bvg phase would probably render them non-essential in that phase. Thus, Bvg-dependent essentiality might be expected to mirror Bvg− regulation patterns. However, surprisingly, this was not the case. None of the conditionally essential genes, or those for which mutation was identified as affecting the fitness of one Bvg phase, were identified as Bvg-regulated (>2-fold change in expression between phases) in an RNAseq-mediated study of gene expression in Bvg+ and Bvg− phase growth (unpublished data and [10]). Thus, conditional essentiality is not due to the differential expression of these genes between Bvg phases, but implies fundamental differences in the contribution of specific genes to *B. pertussis* physiology, dependent on Bvg.

Here, we identify that the process of cell wall biosynthesis can proceed differently between Bvg phases, as Bvg+ phase growth can tolerate the loss of a range of key cell wall biosynthesis genes. We have identified a number of specific genes involved in this difference, providing targets for further study of the phenomenon. Our findings suggest that Bvg not only regulates virulence in *B. pertussis *, but that it coordinates much wider aspects of *B. pertussis* physiology, and understanding the coordination between growth physiology and virulence will be important to fully dissecting *B. pertussis *–host interactions. In addition, *B. pertussis* is able to maintain normal growth rates in the absence of seemingly key cell wall biosynthesis genes, and is able to do this without mutation or acquisition of novel genes. We suggest that understanding mechanisms by which bacteria may withstand disruption of cell wall biosynthesis, a key target of important antibiotics, is important to understanding the mechanisms of antibiotic resistance and for developing effective new antimicrobial drugs.

## Supplementary Data

Supplementary material 1
